# Small Molecule Inhibitors of Microenvironmental Wnt/β-Catenin Signaling Enhance the Chemosensitivity of Acute Myeloid Leukemia

**DOI:** 10.3390/cancers12092696

**Published:** 2020-09-21

**Authors:** Paul Takam Kamga, Giada Dal Collo, Adriana Cassaro, Riccardo Bazzoni, Pietro Delfino, Annalisa Adamo, Alice Bonato, Carmine Carbone, Ilaria Tanasi, Massimiliano Bonifacio, Mauro Krampera

**Affiliations:** 1 Section of Hematology, Stem Cell Research Laboratory, Department of Medicine, University of Verona, 37134 Verona, Italy; Paul.Takam-Kamga@ac-versailles.fr (P.T.K.); g.dalcollo@erasmusmc.nl (G.D.C.); riccardo.bazzoni@univr.it (R.B.); annalisa.adamo@univr.it (A.A.); alice.bonato@icgeb.org (A.B.); ilaria.tanasi@univr.it (I.T.); massimiliano.bonifacio@univr.it (M.B.); 2 EA4340-BCOH, Biomarker in Cancerology and Onco-Haematology, Université de Versailles-Saint-Quentin-En-Yvelines, Université Paris Saclay, 92100 Boulogne-Billancourt, France; 3 Department of Immunology, Erasmus University Medical Center, Doctor Molenwaterplein 40, 3015 GD Rotterdam, The Netherlands; 4 Department of Oncology, Hematology Unit, Niguarda Hospital, 20162 Milan, Italy; adriana.cassaro@ospedaleniguarda.it; 5 Department of Health Sciences, University of Milan, 20146 Milan, Italy; 6 Department of Diagnostics and Public Health, University and Hospital Trust of Verona, 37134 Verona, Italy; pietro.delfino@univr.it; 7 Fondazione Policlinico Universitario Gemelli, IRCCS, 00168 Roma, Italy; carmine.carbone@univr.it

**Keywords:** microenvironment, Wnt, AML, drug target

## Abstract

**Simple Summary:**

Considering the pivotal role of Wnt/β-catenin signaling in AML development and persistence, the current study addresses in AML, the prognostic value of Wnt/β-catenin signaling molecules and the anti-leukemic value of Wnt/β-catenin inhibition. In silico analysis of RNAseq data from AML patients and flow cytometric analysis of primary AML samples revealed that higher levels of Wnt/β-catenin pathway is a poor prognostic marker. Next, we found that pharmacological interference, through small molecule inhibitors of Wnt and/or GSK-3 signaling reduces AML cell survival by sensitizing the leukemia cells to chemotherapeutic agents both in vitro and in vivo. Overall, our findings suggested that Wnt-inhibitory therapy could overcome the prognostic significance of patient risk stratification, standing as a therapeutic response for all subgroups of AML.

**Abstract:**

Wnt/β-catenin signaling has been reported in Acute Myeloid leukemia, but little is known about its significance as a prognostic biomarker and drug target. In this study, we first evaluated the correlation between expression levels of Wnt molecules and clinical outcome. Then, we studied—in vitro and in vivo—the anti-leukemic value of combinatorial treatment between Wnt inhibitors and classic anti-leukemia drugs. Higher levels of β-catenin, Ser675-phospho-β-catenin and GSK-3α (total and Ser 9) were found in AML cells from intermediate or poor risk patients; nevertheless, patients presenting high activity of Wnt/β-catenin displayed shorter progression-free survival (PFS) according to univariate analysis. In vitro, many pharmacological inhibitors of Wnt signalling, i.e., LRP6 (Niclosamide), GSK-3 (LiCl, AR-A014418), and TCF/LEF (PNU-74654) but not Porcupine (IWP-2), significantly reduced proliferation and improved the drug sensitivity of AML cells cultured alone or in the presence of bone marrow stromal cells. In vivo, PNU-74654, Niclosamide and LiCl administration significantly reduced the bone marrow leukemic burden acting synergistically with Ara-C, thus improving mouse survival. Overall, our study demonstrates the antileukemic role of Wnt/β-catenin inhibition that may represent a potential new therapeutics strategy in AML.

## 1. Introduction

Acute myeloid leukemia (AML) is the most common acute leukemia in adults; relapse after chemotherapy still represents a critical problem in most patients, with only a 35–40% five-year overall survival rate for non-promyelocytic AML [1,2]. Nevertheless, accurate biomarkers for early diagnosis and response prediction to chemotherapy or allogeneic transplantation are still lacking [3]. The persistence of residual leukemia cells during chemo- or immunotherapy is favored by different components of the bone marrow stromal niche through a redundant panel of molecular pathways, such as Wnt, Notch and Hedgehog, supporting cell survival, proliferation and chemoresistance [4,5,6]. However, the role of these pathways in the interaction between AML cells and bone marrow microenvironment is not entirely clear yet [7,8].

Wnt/β-catenin signaling is a developmental pathway mostly involved in tissue patterning during embryonic development, through its ability to modulate proliferation, differentiation and motility of embryonic and adult stem cells [9]. The pathway is activated when secreted Wnt proteins (Wnt-1, Wnt-3a, Wnt-3b etc.) bind to the extracellular domain of the frizzled family of receptors and lipoprotein receptor-related protein (LRP) co-receptors. This binding stabilizes β-catenin by disrupting the β-catenin destruction complex consisting in GSK-3 and other proteins. The inactivation of the β-catenin destruction complex promotes the nuclear accumulation of β-catenin, which in turn triggers the expression of several target genes [10]. Deregulation of the pathway can occur in cancer, either as result of gene mutations in components of Wnt/β-catenin pathway or because of the crosstalk between tumor-associated stroma and cancer cell bulk. This deregulation can induce a persistent accumulation of β-catenin in the nucleus, thus favoring cancer growth and dissemination, maintenance of cancer stem cells, and drug resistance [11,12]. Considering the pivotal role of Wnt/β-catenin signaling in several cancers, small molecules inhibitors have been developed as potent anticancer agents, including inhibitors of Frizzled, Porcupine, GSK-3 and disruptors of the β-catenin/TCF/LEF complexes [13].

In AML, a number of studies support the contribution of the canonical Wnt signaling in the maintenance of leukemia stem cells (LSCs) [14]. Indeed, many molecular events contribute to the aberrant expression of the Wnt pathway in AML cells, such as FLT3 signaling, hypermethylation of the secreted frizzled-related proteins, high levels of Frizzled receptors and Wnt ligands [15]. Moreover, preclinical studies have demonstrated that activating the mutation of β-catenin in stromal cells is sufficient to induce AML-like disease in mice [16]. Overall, the canonical Wnt signaling appears relevant for leukemogenesis in AML and therefore drug targetable.

In this work, we demonstrate that pharmacological interference, through small molecules inhibitors of Wnt and/or GSK-3 signaling reduces AML cell survival by sensitizing the leukemia cells to chemotherapeutic agents both in vitro and in vivo. Consequently, combination treatments with small molecules inhibitors of Wnt/GSK3 axis and chemotherapy may represent a novel therapeutic strategy for a better chance of AML cell eradication.

## 2. Materials and Methods

### 2.1. Chemicals and Antibodies

The antibodies used for blast cell identification by Flow Cytometry (FACSCanto II, Becton Dickinson, Rutherford, NJ, USA) were: anti-CD45-VioBlue, anti-CD45-APC-Vio770, anti-CD34-PerCP and anti-CD117-APC all from Miltenyi Biotech (Bergsch gradbach, Germany). For Western blot analysis, anti-β-catenin, anti- Ser675-phospho-β-catenin, anti-Ser33/37/Thr41-phospho-β-catenin, anti-non-phopho-β-catenin, anti-GSK-3β, anti-Ser9-phospho-GSK-3β, anti-GSK-3α, anti-Ser21-phospho-GSK-3α anti-Histone H3 antibodies and Alexa 488-conjugated secondary antibodies were from Cell Signaling (Danvers, MA, USA); anti-GAPDH, and HRP-conjugated secondary antibodies against mouse or rabbit were from Sigma-Aldrich (Darmstadt, Germany). Wnt modulators used for proliferation and vitality assays, i.e., Wnt-3a, PNU-74654, Niclosamide, IWP-2, Lithium Chloride (LiCl), and AR-A014418, were all purchased from Sigma-Aldrich. For the analysis of cell death, Propidium iodide (PI) and FITC-conjugated Annexin V were from Miltenyi Biotechnology. CellTiter 96^®^ AQ_ueous_ One Solution Cell Proliferation Assay (MTS, Eden Prairie, MN, USA) was from Promega (Promega, Milano, Italy). Cytarabine (Ara-C) and Idarubicin (Ida) were provided by the Pharmacy Unit of the University Hospital of Verona.

### 2.2. Patients, Samples and Cell Lines

All cell samples were collected from AML patients and healthy donors after written informed consent, as approved by the Ethics Committee of Azienda Ospedaliera Universitaria Integrata Verona (N. Prog. 1828, 12 May 2010—‘Institution of cell and tissue collection for biomedical research in Onco-Hematology’). In detail, AML blast cells were obtained from bone marrow or peripheral blood samples of patients with AML at diagnosis (>80% of leukemia cells). Samples with less than 80% of blast cells were enriched with CD34^+^ using MACS CD34 Microbead Kit (MiltenyiBiotec). AML patients characteristics have been described elsewhere [17] and summarized in Table S1. Human bone marrow mesenchymal stromal cells were obtained from bone marrow aspirates of healthy donors (hBM-MSCs, *n* = 12) and AML patients (hBM-MSCs*, *n* = 18) after informed consent as previously described [17,18]. Human cell lines HL-60 (acute promyelocytic leukemia cell line), THP1 (acute monocytic leukemia cell line), U937(myeloid histiocytic sarcoma cell line), were grown in complete RPMI-1640 medium (RPMI supplemented with 10% FBS, 1% L-Glutamine solution 200 mM and 1% Penicillin/Streptomycin). HEK-293 (human embryonic kidney cell line) and hBM-MSCs were maintained in complete DMEM. Cell lines were purchased from the American Type Culture Collection. Flow cytometry of membrane marker and cell morphology through Giemsa staining were used to check stability and identity of cell lines as previously described [19]. Cell lines were routinely tested to be Mycoplasma-free.

### 2.3. Western Blotting

Immunoblotting were performed as previously described [19]. Briefly, Cells were lysed with the RIPA lysis buffer (25 nM Tris pH 7.6, 150 mM NaCl, 1% NP40, 1% Na-deoxycholate, 0.1% SDS). Then, samples were subjected to SDS-PAGE (sodium dodecyl sulfate polyacrylamide gel electrophoresis) followed by protein transfer onto nitrocellulose membrane (GE Healthcare, Chicago, IL, USA), that were subsequently probed with antibodies specific to target proteins.

### 2.4. Cell Proliferation and Apoptosis and Viability Assays

The IC50 for each drug were obtained by analyzing treated cells with the colorimetric One Solution Cell Proliferation Assay (MTS), as previously described [19,20]. Cell proliferation, cell death and apoptosis were assessed through flow cytometric analysis of AML cells stained with CFSE (carboxyfluorescein succinimidyl ester, proliferation) TOPRO-3 (cell death) and FITC-Annexin V/Propidium Iodide (PI) (apoptosis) as previously described [17,18,19].

### 2.5. Xenograft Mouse Model

Animal care was performed in accordance with institution guidelines as approved by the Italian ministry of health. Mice were purchased from Taconic (Germantown, NY, USA). Animal experiments were carried in pathogen-free conditions at the animal facility of the Interdepartmental Centre of Experimental Research Service of the University of Verona. Parameters used for sample size are power of 80%, a signal/noise ratio of 2 and a significance level of 5% (*p* ≤ 0.05) using a one-sample *t*-test power calculation. With a commitment to providing refinement, reduction and replacement (3Rs), application of factorial design to reduce the minimum group size was applied to obtain a minimum group size of 5–8 mice. Xenograft mouse model were generated in NOD/Shi-scid/IL-2Rγnull (NOG, Minnetonka, MN, USA) mice as previously described [17]. Briefly, U937 AML cell line (1 × 10^6^) were injected into the tail vein of totally irradiated (1.2 Gy, ^137^Cesium source), 8–12-week-old male mice. At day 9 post-injection, mice (randomly allocated) received for 5 days intraperitoneal daily injection of Ara-C (25 mg/Kg) or its vehicle (dimethyl sulfoxide, DMSO). In case of combined treatment, Ara-C or DMSO were associated with each Wnt/GSK-3 inhibitor for the first two days, followed by three days of Ara-C or DMSO. Mice were sacrificed after 2 weeks following the cell line injection, and bone marrow leukemic burden was evaluated as percentage of human hCD45+ cells. No blinding was done. Wnt inhibitors used for mouse experiments were PNU-74654 (0.5 mg/kg), Niclosamide (10 mg/Kg) and LiCl (25 mg/Kg).

### 2.6. Cell Culture and Co-Culture

To study the capability of hBM-MSCs to support AML cell survival, AML cells were cultured in suspension or on a confluent monolayer of hBM-MSCs with complete RPMI 1640 in 96-well plates: 10^5^ AML blast cells or 2 × 10^4^ cells from AML cell lines were cultured in 100 μL of complete RPMI for 48 h. Wnt modulators, Ara-C and Idarubicin were added in co-culture. Co-cultured AML cells were collected and stained with anti-CD45 antibodies conjugated to APC or PerCP and analyzed for proliferation (CFSE dilution) and cell death (TOPRO-3, Annexin V/PI).

### 2.7. Flow Cytometry Analysis of Wnt Molecules

AML cell lines or AML blast cells identified as CD45+, CD34+, CD38- cells by flow cytometry, were fixed and permeabilized for 30 min at 4 °C. Permeabilized cells were probed with primary antibodies or their specific isotype for 1 h. Subsequently cells were washed and labelled for 30 min with Alexa 488-conjugated secondary antibodies. Protein expression was then analyzed through flow cytometry and expressed as relative median of fluorescence intensity (rMFI), defined as the ratio of the specific antibody fluorescence over the specific isotype fluorescence.

### 2.8. Gene Reporter

To monitor Wnt/β-catenin transcriptional activity, we transfected THP1 with reporter plasmids encoding for an inducible TCF responsive GFP reporter (Qiagen, Hilden, Germany) as previously described [8] GFP signal was observed through Axiovert Z1 (Zeiss, Sheung Kehen, Germany) and quantitatively measured by flow cytometry. The Wnt pathway activity was determined by normalizing the activity of TCF-GFP to that of CMV-GFP plasmid.

### 2.9. RNA-seq Analysis

RNA-seq data from 173 AML patients were obtained as part of The Cancer Genome Atlas project (TCGA). Gene expression data in Z-score format were downloaded from the cbioportal R package cgdsr for the TCGA-LAML. Normalized data were used for Z-score calculation. For each mRNA, a sample showing a Z-score higher or lower than the average Z-score for the whole population was considered as expressing highly or lowly, respectively, the corresponding gene, Z > +/− 1.96; *p* < 0.05. This strategy was applied for the gene of the WNT signaling pathways (APC, AXIN1, CTNNB1, FZD4, GSK3A, GSK3B, LRP5, TCF4, WNT3A, WNT5A, WNT5B, WNT10A, WNT10B).

### 2.10. Statistical Analysis

Statistical analysis was performed using GraphPad Prism software (La Jolla, CA, USA). Mann–Whitney and Kruskal–Wallis were used to compare two groups or more than two groups, respectively. All tests were one-sided. Pearson Chi-square analysis was used to test association among variables. Survival curves were calculated by the Kaplan–Meier Method.

## 3. Results

### 3.1. Wnt/GSK-3 Axis Is Functional in AML Cell Lines

**Figure 1:**
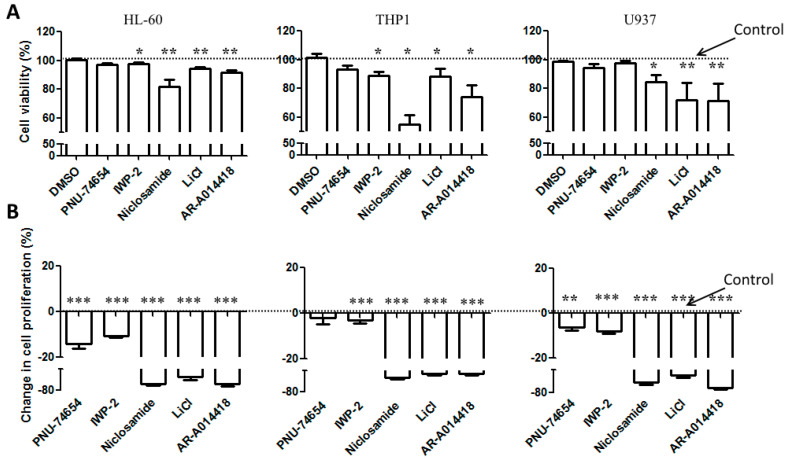
Cell viability and proliferation of AML cell lines cultured in the presence of Wnt and GSK-3 inhibitors. AML cells lines were stained with CFSE and cultured in the presence of either Wnt modulators, including PNU-74654 (15 µM), IWP-2 (15 µM) Niclosamide (1 µM), or GSK-3 inhibitors, including LiCl (10 mM), AR-A014418 (15 µM). After 4 days, cells were stained with TOPRO-3 to exclude dead cells. (**A**) Viable cells (TOPRO-3 negative cells) and CFSE-stained cells were quantified by FACS analysis. (**B**) Relative cell proliferation was expressed as the percentage of CFSE median fluorescence of treated cells compared to the cells treated with DMSO. Data are reported as mean ± SEM of 5 independent experiments. A Mann–Whitney test was used to analyze the differences between means. * *p* < 0.05, ** *p* < 0.01, *** *p* < 0.001. Dot line: control.

We first evaluated in three AML cell lines, HL-60, THP1 and U937, the basal expression and activation of the Wnt molecules, including total β-catenin, pan-phosphorylated β-catenin(Ser33–37/Thr41), Ser675-phospho-β-catenin, active non-phospho-β-catenin, GSK-3β (total and Ser9) and GSK-3α (total and Ser9). These proteins were expressed in all the three cell lines (Table 1). Western immunoblot of nuclear fraction confirmed the activation of the Wnt/β-catenin pathway, since β-catenin was found in the nuclear fraction of lysate for each cell line (Figure S1A). Next, we used a pharmacological approach to confirm the activation of the pathway in AML cell lines by adding Wnt inhibitors (PNU-74654, IWP-2 and Niclosamide) or GSK-3 inhibitors (LICL, AR-A014418) in the culture medium. Cells were treated with increasing concentrations of each inhibitor, then cell viability was assessed through MTS. The samples treated with all the GSK-3 and Wnt inhibitors, except IWP-2, displayed reduction in cell viability in a dose-dependent manner (Figure S1B). As MTS assay cannot discriminate cell death or cell proliferation, cell lines were treated with a single concentration (close to the IC50) of each drug, and then cell death and proliferation were analyzed using TOPRO-3 and CFSE staining. Except Niclosamide, which induced a strong effect, the other Wnt inhibitors PNU-74654 and IWP-2 slightly reduced cell viability, while inducing a significant reduction in cell proliferation (Figure 1A,B). GSK-3 inhibitors, including LiCl and AR-A014418, induced a significant reduction in both cell viability and proliferation (Figure 1A,B) in all the tested cell lines. THP1 cell line, expressing higher levels of Wnt proteins compared to the two other cell lines (Table 1 and Figure S1A,B), was mostly sensitive to Wnt inhibitors (Figure 1A). The use of Wnt ligands, such as Wnt-3a and Wnt-5a, did not modify either cell proliferation or cell survival (Figure S1C).

### 3.2. Wnt Molecules Are Enriched in Patient Samples

**Figure 2:**
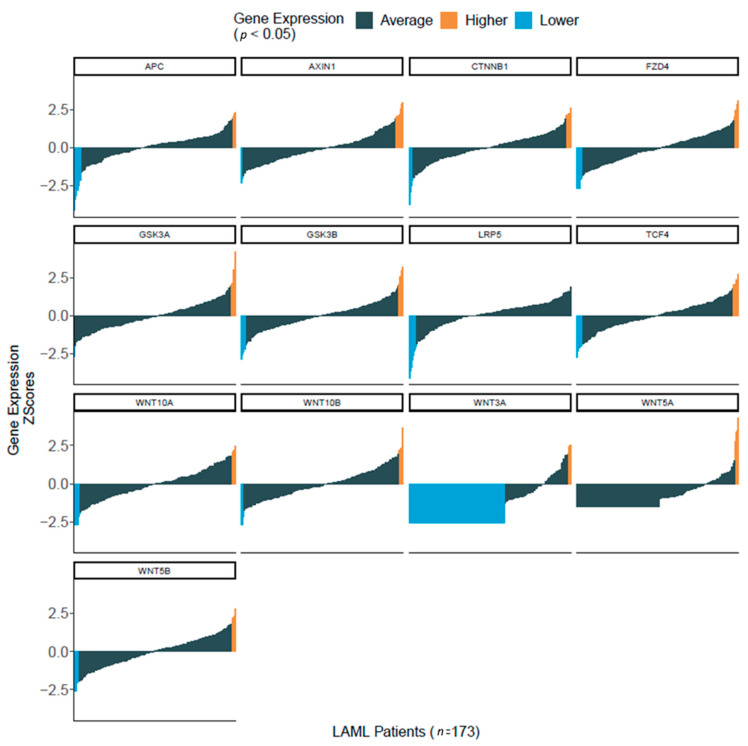
Enrichment of Wnt signaling component in primary AML samples. Wnt signaling expression data from 173 de novo AML samples obtained from The Cancer Genome Atlas RNA-Seq database for AML, Gene expression data in Z-score format were downloaded from the cbioportal R package cgdsr for the TCGA-LAML. Normalized data were used for Z-score calculation. For each mRNA, a sample showing a Z-score higher or lower than the average Z-score for the whole population was considered as having higher or lower expression respectively for the corresponding gene, Z +/− 1.96 (*p* < 0.05).

**Figure 3:**
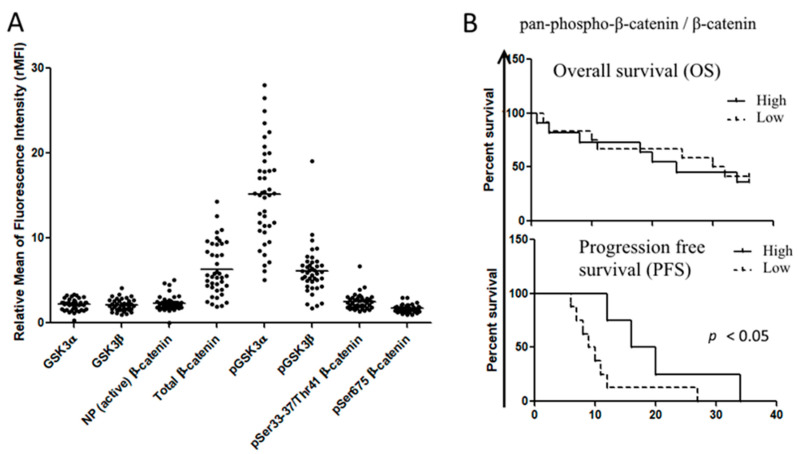
Expression of Wnt signaling in primary AML samples. (**A**) Flow cytometric analysis of Wnt components in AML primary samples (*n* = 60), probed with specific primary antibody and labeled with FITC-conjugated secondary antibodies. Data were expressed as relative median of fluorescence intensity. (**B**) The ratio between inactive Ser33–37/Thr41-phospho-β-catenin and total β-catenin was evaluated for each sample and classified as high ratio or low ratio when they were above and below the mean ratio value for all samples, respectively. Gehan–Breslow–Wilcoxon analysis was used to establish the difference in overall survival and progression-free survival among the groups.

**Figure 4:**
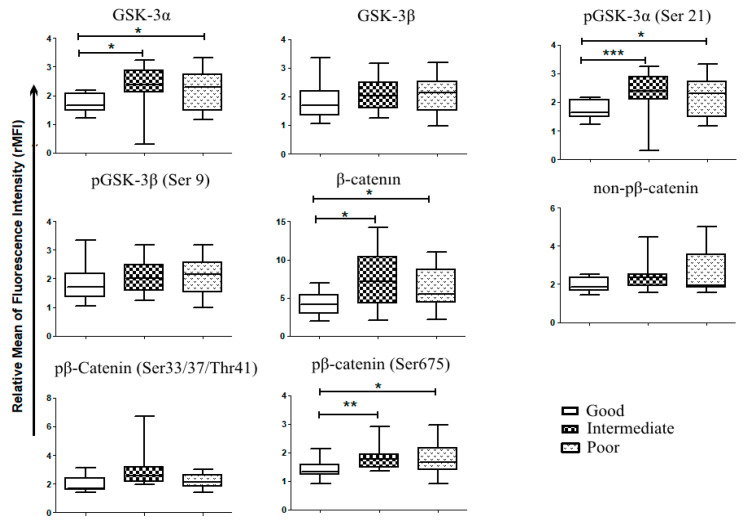
Wnt and GSK-3expression in primary AML samples according to patient stratification. Patient samples analyzed for Wnt expression, were stratified according to their mutational status and their cytogenetics into 3 risks group as proposed by the European Leukemic Network (ELN)s; favorable risk or good (*n* = 11), intermediate (*n* = 12) and poor (*n* = 13). Mann–Whitney test was used to analyze differences between the two groups * *p* < 0.05, ** *p* < 0.01, *** *p* < 0.001.

To determine whether the Wnt/β-catenin pathway was represented in patient samples, we first used in silico analysis of RNA-seq data from more than 170 AML patients that were part of The Cancer Genome Atlas (TCGA) project on AML [21]. We observed that several genes of the Wnt/GSK-3/β-catenin axis were enriched in AML samples, including *AXIN*, *APC*, *CTNNB1* (BETA-CATENIN), *GSK-3A* and *GSK-3B* (Figure 2). We then used flow cytometric analysis to determine the Wnt expression pattern in a cohort of 60 AML patients admitted to our institution. Consistently with in silico analysis, we observed in primary AML samples, a robust expression of β-catenin (total and phosphorylated forms), GSK-3β (total and Ser9) and GSK-3α (total and Ser21) (Figure 3A). However, the expression levels of each protein were not homogeneous amongst samples. To investigate whether this heterogeneous expression of Wnt molecules could have prognostic significance, the samples were classified according to the expression degree (high versus low) as compared to the mean values of expression for all samples. Then, we used a Kaplan–Meier analysis to determine patient survival in the two groups, censoring data after 36 months. For all the proteins considered, no significant differences were observed neither in overall survival (OS), nor in progression free survival (PFS). By contrast, when we considered the activation of β-catenin pathway as the ratio between the non-active form (Ser33/37/Thr41 β-catenin) and the total β-catenin, patients with low activation status (high ratio) displayed a better PFS compared to patients presenting high activation status of the pathway (low ratio) (Figure 3B). Pearson analysis showed a positive association between leucocyte count (WBC) and expression levels of pan-phospho-β-catenin (Ser33–37/Thr41), Ser 675-phospho-β-catenin, non-phospho-β-catenin and GSK-3β (Table 2A). A positive association was also found between hemoglobin (Hb) level and active β-catenin (non-phosphorylated), phospho-GSK-3β (Ser 9), pan-phospho-β-catenin and Ser 675-phospho-β-catenin) (Table 2B). The European Leukemia Network (ELN) recommendations for diagnosis and management of AML in adults has proposed the stratification of AML patients according to genetic and molecular characteristics, dividing patients into three risk categories that are relevant for clinical outcomes, i.e., good/favorable, intermediate, poor/adverse [22]. Accordingly, thanks to the cytogenetics and mutational pattern of each patient, we classified patients in these three risk groups, observing that β-catenin, Ser675-phospho-β-catenin and GSK-3α (total and Ser21) were preferentially expressed in adverse and intermediate risk groups (Figure 4). All these observations suggested that Wnt or GSK signaling could be associated with AML chemosensitivity.

### 3.3. hBM-MSCs Express Wnt Molecules but are Insensitive to Pathway Inhibitors

**Figure 5:**
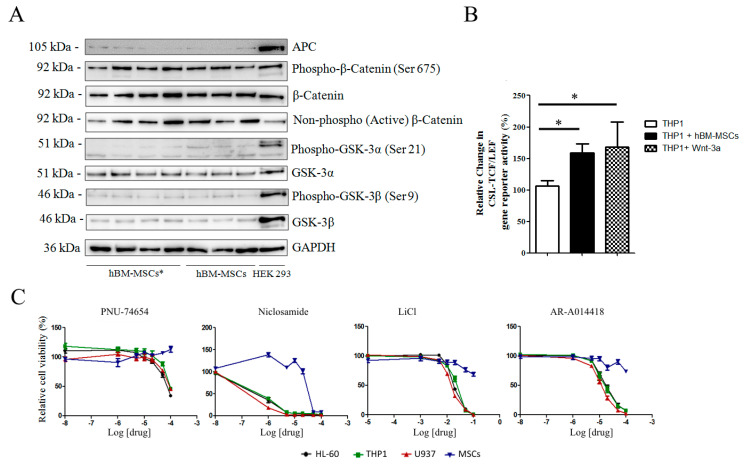
Expression of Wnt and GSK-3 molecules in hBM-MSCs. (**A**) Representative Western blot analysis (Figure S3) of Wnt components in AML-hBM-MSC (hBM-MSCs *) and hBM-MSCs from normal donors. Images are representative of 12 hBM-MSC and 18 hBM-MSC * samples. (**B**) Wnt activity according to GFP signal in THP1 cells expressing the gene reporter CSL-TCF/LEF-GFP. Transfected cells were cultured either alone or in presence of hBM-MSCs or in medium supplemented with Wnt-3a (25 ng/mL). (**C**) hBM-MSC viability in growth medium supplemented with increasing concentrations of Wnt and GSK-3 inhibitors. Data are representative of at least 4 independent experiments. * *p* < 0.05.

Considering the importance of bone marrow microenvironment in AML onset and recurrence, ex-vivo co-culture of leukemic cells on bone marrow stromal monolayer represents a good tool for evaluating drug sensitivity [23,24]. Bone marrow stromal cells from healthy donors and patients were analyzed for expression of components of Wnt/GSK-3/β-catenin axis. Western blot analysis highlighted that all healthy donors hBM-MSCs (*n* = 12) and AML-hBM-MSCs (hBM-MSCs*, *n* = 18) samples expressed Wnt components, suggesting a possible paracrine signal between hBM-MSCs and leukemic cells. The presence of active forms of β-catenin, including non-phospho-β-catenin and Ser675-phospho-β-catenin [25], revealed a constitutive activation of the Wnt/β-catenin pathway in these cells (Figure 5A). To assess whether hBM-MSC and AML cell co-culture induces the activation of Wnt/β-catenin in AML cells, we used THP1 cells expressing the TCF/LEF-GFP reporter gene. Transfected cells seeded on hBM-MSCs displayed enhanced GFP signal (Figure 5B) and the increase in TCF/LEF activity was similar to that observed when cells were challenged with Wnt-3a (Figure 5B). As the inhibition of the pathways could theoretically interfere with some hBM-MSC functions, we assessed hBM-MSC viability through MTS assay with increasing concentrations of Wnt/GSK-3 inhibitors. hBM-MSCs and hBM-MSCs* viability was not altered by Wnt/GSK-3 inhibitors unless at very high concentrations (Figure 5C). We also analyzed immunomodulatory properties and adipogenic or osteogenic differentiation of hBM-MSCs treated with Wnt or GSK-3 inhibitors for 48 h; hBM-MSCs retained the same capability both to suppress stimulated PBMC proliferation (Figure S2A) and to undergo differentiation into osteocyte and adipocytes (Figure S2B).

### 3.4. Wnt Modulators Enhance Chemosensitivity of AML Cells

**Figure 6:**
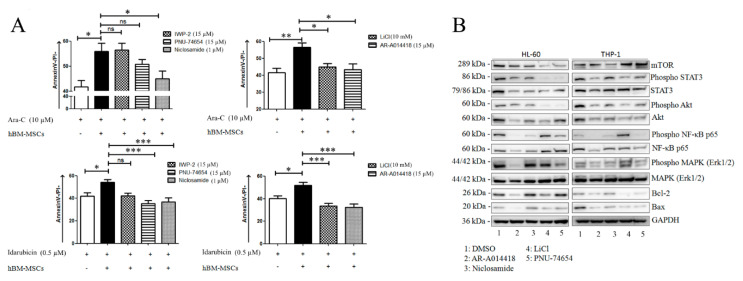
Wnt modulators improve chemosensitivity of AML blasts. (**A**) Primary AML blasts were treated with Ara-C or Idarubicin in presence or absence of hBM-MSCs and Wnt or GSK-3 inhibitors. After 48 h of incubation, cells were collected and stained with AnnexinV/PI to evaluate cell apoptosis. Data are expressed as mean ± SEM of at least 3 independent experiments performed in triplicate: * *p* < 0.05, ** *p* < 0.01, *** *p* < 0.001, ns (not significant). (**B**) Representative Western blot analysis (Figure S5) of HL-60 and THP1 cell lines treated for 48 h with Wnt or GSK-3 inhibitors, including PNU 74,654 (15 µM), IWP-2 (15 µM), Niclosamide (1 µM), LiCl (15 mM) and AR-A014418 (15 µM). Images are representative of at least 3 experiments.

We assessed whether Wnt inhibitors could increase AML cell sensitivity to drugs normally used for AML therapy, such as Idarubicin or Ara-C. AML cells were cultured alone or in the presence of hBM-MSCs for 48 h, with or without Wnt modulators and chemotherapeutic agents. Cells were harvested, stained with annexin and analyzed for apoptosis. Idarubicin (0.5 µM) or Ara-C (10 µM) significantly induced the apoptosis of both AML cell lines and AML primary cells, while co-culture with hBM-MSCs led to a significant rescue effect (Figure 6A and Figure S4). The addition of PNU-74654 (15 µM), Niclosamide (1.5 µM), LiCL (15 mM) or AR-A014418 (15 µM) significantly lowered the anti-apoptotic effect on AML cells mediated by bone marrow stromal cells, regardless their origin (hBM-MSCs or hBM-MSCs*) and the ELN risk classification of the AML patients.

To investigate which microenvironmental prosurvival protein network was affected by Wnt pharmacological inhibition, we first analyzed how the Wnt-GSK axis was modulated in AML cell lines treated with above mentioned inhibitors, observing that the pattern of modulation was cell line-dependent, probably reflecting differential modulation of the pathway according to molecular features of the AML cell/samples (Figure S6). Briefly, Niclosamide and PNU-74654 reduced the levels of Ser675-phospho-β-catenin in the HL-60 cell line, whereas Niclosamide induced reduced levels of both total β-catenin and the pan-phospho-β-catenin in the THP1 cell line. Of note, as previously described, the PNU-74654 treatment induced increased levels of total β-catenin [26]. This effect could be related to the accumulation of the protein in the cytoplasm, because the inhibition of the Tcf/β-catenin complex, PNU-74654, decreased nuclear β-catenin accumulation (Figure S6A) [26]. In contrast, both Wnt and GSK-3 inhibitors were associated with reduced phosphorylation of GSK-3β at Ser 9 (Figure S6B). Then, we analyzed the expression and activation of Bcl-2, mT/Akt and MAP kinase family proteins in THP1 and HL-60 cell lines treated with above mentioned inhibitors. A persistent modulation of Bax, STAT-3, Akt, NF-κB, and ErK 1/2 was clearly evident (Figure 6B).

**Figure 7:**
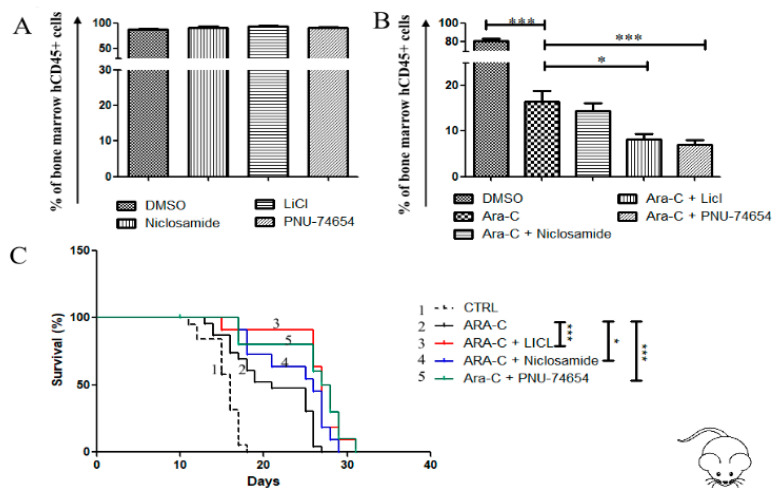
Wnt inhibition reduces bone marrow leukemic burden and prolongs survival of the cell line-based AML xenograftmouse model. (**A**,**B**) Flow cytometry analysis of human CD45+ in bone marrow samples obtained from mice transplanted with AML cell line U937. Starting from day 9 post-engraftment, mice were treated for 2 days with one of the following schedules: DMSO, Ara-C alone (25 mg/Kg), Wnt inhibitor alone, or Wnt inhibitor + Ara-C. Then Ara-C were administered for further 3 days in the groups receiving Ara-C (alone or in combination), while the other groups received DMSO. The following concentrations were used for each inhibitor: PNU-74654 0.5 mg/kg, Niclosamide 10 mg/kg, LiCl 25 mg/kg. The assay was performed with at least 5 mice in each group. Data are reported as mean ± SEM of values obtained from at least 5 mice. (**C**) Survival of mice transplanted with U937, differences in survival curves were analyzed with the Log-rank (Mantel–Cox) Test, * *p* < 0.05, *** *p* < 0.001.

To assess whether he anticancer effect of Wnt and GSK-3 inhibitors could be operational in vivo, we generated a xenograft model of AML by injecting the U937 cell line in the tail vein of NOG mice, as previously described [17]. Two weeks later, the mice were treated with either Ara-C alone or Ara-C in combination with each inhibitor (except AR-A014418 for its known toxicity). Treatment of engrafted mouse with Ara-C significantly reduced the leukemic burden in mouse bone marrow (Figure 7A), but this effect was stronger when Ara-C was associated with either PNU-74654, Niclosamide, or LiCl. Accordingly, the mean of mouse survival was significantly improved when Ara-C was associated with each inhibitor, i.e., PNU-74654 (33 days), Niclosamide (29 days) and LiCl (33 days), as compared to mice treated with Ara-C alone (mean survival = 26 days) (Figure 7B).

## 4. Discussion

Targeted treatments using small molecule inhibitors are extensively studied as promising strategies to eradicate drug resistance in cancer and in AML [27]. In this study, we demonstrated—in vitro, ex vivo and in vivo in a murine model—that the use of pharmacological inhibitors of Wnt/β-catenin enhances the effectiveness of chemotherapeutic agents in eradicating AML cells.

The rationale for using pharmacological inhibitors of Wnt signaling as anti-leukemic agents is the evidence of aberrant activation of Wnt pathway in AML samples [28,29]. Through flow cytometric analysis, we confirmed that β-catenin was present in AML primary cell samples in a variable phosphorylated status, including the Ser675-phospho-β-catenin and the (Ser33/37/Thr41)-phospho-β-catenin. Considering the heterogeneity of each AML sample, we did not use Western blot of nuclear fraction as a method to analyze Wnt activation in samples. We took the (Ser33/37/Thr41)-phospo-β-catenin/total β-catenin ratio as a surrogate for assessing Wnt/β-catenin activation, demonstrating that the pathway is constitutively active in a large portion of patients. Besides the robust expression of β-catenin, we also observed a robust expression of different forms of GSK-3 proteins in AML samples. Within the Wnt/β-catenin cascade, GSK-3 is considered as a negative regulator of the pathway [30,31]. Its inhibition is often used as an indirect strategy to stabilize cellular β-catenin [32,33]. Nevertheless, we report here that the use of GSK-3 inhibitors reduced cell viability, similarly to Wnt inhibition. There are at least two pools of GSK-3, one associated with Axin and involved in β-catenin phosphorylation, the other not associated with Axin. Interestingly, Wnt signaling can modulate both β-catenin-associated GSK-3 and β-catenin-independent GSK-3 [30,34]. Evidence from the literature also shows that GSK-3 is not only involved in the Wnt signaling, but also crosstalk with multiple others survival pathways, such as Notch, Hegehog and Akt-related cascades [27,32,34,35,36,37,38]. As the role of GSK-3 cannot be only related to Wnt/β-catenin signaling, we cannot exclude the involvement of a GSK-3-independent Wnt signaling in AML pathogenesis [27,32,34,35,36,37,38]. Interestingly, a previous work has demonstrated that GSK-3 inhibition can improve 1,25-dihydroxyvitamin D3-mediated differentiation of AML cells, suggesting that a combinatorial treatment including GSK-3 inhibitors and anti-leukemic drugs is a promising and safety strategy in AML [39].

Wnt/β-catenin signaling is a multiple step cascade. The importance of each step for cancer development is disease- and cell context-dependent [40,41]. The inhibitors used in this study were chosen for their ability to interfere with specific steps of the pathway, i.e., ligand and receptors (IWP-2 and Niclosamide), GSK-3 (LiCl, AR-A014418), or nuclear transactivation or TCF/LEF complex (PNU-74654 and PKF118-310). While all GSK-3 inhibitors and TCF/LEF inhibitors showed the same activity for each member of Wnt family, IWP-2 failed to reproduce the capability of Niclosamide of reducing AML cell viability and sensitizing them to drugs. This discrepancy can be explained by the mechanisms triggered by each of the inhibitors; in fact, IWP-2 interferes with biogenesis of Wnt ligand, by targeting porcupine, while Niclosamide interferes directly with the co-receptor LRP-6 [13,42].

Our data show that small molecule inhibitors of Wnt or GSK-3 substantially enhance the antitumor effect of Ara-c and Idarubicin in vitro and reduce AML cell survival advantage, thus lowering the leukemic burden in the bone marrow of AML mouse models. The mechanisms involved in Ara-C or Idarubicin-induced cell death include the modulation of many pathways, such as mTor/Akt, Erk, NF-KB, stat3, Bax/Bak etc [17,18,43,44,45]. Through Western blot analysis, we clearly showed that these proteins were down-regulated in AML cell lines treated with Wnt or GSK-3 inhibitors. These observations are in favor of a synergistic activity between our inhibitors and the chemotherapeutic agents used in this study. We cannot exclude the interaction with other specific mechanisms of drug resistance, but Wnt/β-catenin signaling seems to play a pivotal role in this phenomenon.

The main molecular events related to Wnt pathway activation in AML are both chromosomal translocations and/or mutations of *FLT3* [15]. Enhanced FLT3 signaling is similarly associated to poor prognosis in AML [22]. However, aberrant Wnt signaling was also observed in patients with normal karyotype, i.e., patients classified as an intermediate-risk group according to WHO and ELN classifications [15,46]. We observed a significant expression of total β-catenin, Ser675-phospo-β-catenin, and GSK-3α (total and Ser 21) molecules in intermediate- and adverse-risk patients compared to favorable-risk groups. In addition, we found a significant association between Wnt/β-catenin activation and PFS, suggesting that Wnt/β-catenin signaling could be related to the persistence of residual leukemic cells along the treatment course. Residual resistant cells are mainly found in the bone marrow, as the result of a close interaction between bone marrow microenvironment and AML blast cells and leukemia stem cells. This phenomenon is associated with drug resistance and relapse [47,48,49]. Several literature data permit to elaborate a model of AML development, according to which Wnt/β-catenin is both involved in the crosstalk between stromal cells and AML cells, and in autocrine signaling amongst AML cells [46,50]. Our co-culture data support this hypothesis, demonstrating through gene reporter assays the presence of enhanced activity of Wnt/β-catenin in AML cells cultured on stromal cell monolayers. In the model of AML leukemogenesis induced by activating mutation in β-catenin, the stromal cell contribution is at least equal to the role played by hematopoietic cells [16]. Thus, Wnt inhibition may sensitize AML cells to therapy by both targeting directly resistant cells and interfering with the stromal cell support towards leukemic cells that is necessary for the persistence and selection of resistant clones [48]. Therefore, Wnt-inhibitory therapy could overcome the prognostic significance of patient risk stratification, standing as a therapeutic response for all subgroups of AML.

## 5. Conclusions

Overall, our study demonstrates that because of their capabilities to interfere with AML cell growth and chemoresistance, small molecule inhibitors of Wnt and GSK-3 signaling may represent potential candidates for drug development in AML.

## Figures and Tables

**Table 1:** Flow cytometric analysis of Wnt/β-catenin and GSK-3 molecules in AML cell lines. HL-60, THP1 and U937 were probed with specific primary antibodies and labelled with Alexa 488-conjugated secondary antibodies. Data are expressed as relative median of fluorescence intensity and reported as mean ± SEM of 6 independent experiments.

Relative Expression of Wnt Molecules in AML Cell Lines	HL-60	THP1	U937
	Relative median of fluorescence intensity (rMFI) ± SEM		
Total β-catenin	2.466 ± 0.238	6.765 ± 1.508	2.781 ± 0.288
Non-phospho-β-catenin	1.676 ± 0.058	2.360 ± 0.209	1.442 ± 0.0677
Ser675-phospho-β-catenin	3.471 ± 0.202	7.847 ± 1.443	3.398 ± 0.566
Ser33/37/Thr41-phospho-β-catenin	2.135 ± 0.119	3.013 ± 0.395	2.232 ± 0.21
GSK-3α	2.275 ± 0.161	2.654 ± 0.298	2.194 ± 0.18
pGSK-3α (Ser21)	9.355 ± 1.641	1.640 ± 2.678	7.901 ± 1.643
GSK-3β	2.217 ± 0.139	2.456 ± 0.293	1.713 ± 0.064
GSK-3β (Ser 9)	4.600 ± 0.416	9.401 ± 3.046	3.935 ± 0.643

**Table 2:** Correlation between hemogram and Wnt signaling expression in AML blast cells. Spearman analysis was used to assess the correlation between Wnt protein levels in blast cells and parameters of the hemogram, including (**A**) white blood cells (WBC); (**B**) Hemoglobin (Hb), and (**C**) platelets (PLTS). Statistical significance when *p* ≤ 0.05.

**(A) White Blood Cells (WBC)**								
WBC	GSK3α	GSK3β	Non-Phospho-β-catenin	Total β-Catenin	Phospho-GSK-3α (Ser 21)	Phospho-GSK3β (Ser 9)	Ser33/37/Thr41-Phospho- β-Catenin	Ser675-Phospho-β-Catenin
**r**	0.1117	0.3236	0.3205	0.2317	0.1109	0.2414	0.4311	0.4106
*p* value	0.2583	0.0271	0.0284	0.0870	0.2597	0.0780	0.0043	0.0064
**(B) Hemoglobin (Hb)**								
Hb	GSK3α	GSK3β	Non-Phospho-β-Catenin	Total β-Catenin	Phospho-GSK-3α (Ser 21)	Phospho-GSK3β (Ser 9)	Ser33/37/Thr41-Phospho- β-Catenin	Ser675-Phospho-β-Catenin
**r**	−0.02664	−0.02471	0.09962	0.002832	0.1640	0.2041	0.1749	0.2092
*p* value	0.4387	0.4431	0.2816	0.4935	0.1696	0.1162	0.1538	0.1104
**(C) Platelets (PLTS)**								
PLTS	GSK3α	GSK3β	Non-Phospho-β-Catenin	Total β-Catenin	Phospho-GSK-3α (Ser 21)	Phospho-GSK3β (Ser 9)	Ser33/37/Thr41-Phospho- β-Catenin	Ser675-Phospho-β-Catenin
**r**	0.2725	0.2478	0.4430	0.1463	0.2366	0.3473	0.3730	0.4452
*p* value	0.0539	0.0726	0.0034	0.1972	0.0824	0.0190	0.0125	0.0033

## Supplementary Materials

The following are available online at https://www.mdpi.com/2072-6694/12/9/2696/s1, Figure S1: Wnt expression and activity in AML cell lines, Figure S2: Differentiation and immunomodulatory capabilities of hBM-MSCs in presence of Wnt and GSK-3 inhibitors, Figure S3: Original Western blot figures (Figure 5), Figure S4: Contribution of Wnt modulators to AML cell lines chemosensitivity, Figure S5: Original Western blot figures (Figure 6), Figure S6: Representative Western blot analysis of HL-60 and THP1 cell lines treated for 48hours with Wnt or GSK-3 inhibitors, Table S1: List of patients and their characteristics.
