# Supplementary material for: Small Molecule Inhibitors of Microenvironmental Wnt/β-Catenin Signaling Enhance the Chemosensitivity of Acute Myeloid Leukemia
Source: Cancers (Basel). 2020 Sep 21;12(9):2696. doi: 10.3390/cancers12092696 (PMC7565567; doi:10.3390/cancers12092696)
Supplement: Supplementary file 1 [file cancers-12-02696-s001.pdf]

Supplemental material

Supplemental methods

Western blotting

Cells were lysed with appropriate amount of RIPA buffer (25 nMTris pH 7.6, 150 mM NaCl, 1% NP40, 1% Na-deoxycholate, 0.1% SDS) supplemented with complete Protease Inhibitor (Roche) and 1 mM Na3VO4. Proteins were separated on a 10% polyacrylamide gel. Subsequently, proteins were transferred onto nitrocellulose membrane (GE Healthcare), labelled with appropriate antibody and acquired by LAS4000 (GE Healthcare) instrument. Differential expression of β-catenin was evaluated in cytoplasmic and nuclear cell fractions using the same primary antibody, anti-β-catenin (1:1000) and NE-PER Kit (Life Technologies) as detection system, according to manufacturer's instructions. GAPDH and Histone H3 were used as loading cytoplasmic and nuclear control, respectively. All samples subjected to immunoblotting contained more than 80% of leukaemia cells.

Osteocyte and adipocyte differentiations

Differentiation assays were performed on MSC confluent monolayer in 24 well-plates. Osteocyte differentiation was induced by using StemMACS OsteoDiff Medium human from Miltenyi Biotechnology. Adipocyte differentiation medium consisted in α-MEM supplemented with 10% heat-deactivated FBS, 1% L-Glutamine, 100 µg/mL IBMX, 1 µM Dexamethasone and 10 µg/mL insulin. Half medium was changed weekly in each well and the experiment lasted 15 days. Cell differentiation was analyzed by specific stainings. For osteocyte differentiation, cells were washed with PBS, fixed during 5 minutes with 4% paraformaldehyde, wash three times with deionized water and stained with 2% Alizarin (Sigma Aldrich). For adipocyte differentiation, differentiated cells were washed with PBS and fixed with 60% isopropanol for 1 minute at room temperature, then stained with Oil-Red-O for 10 minutes at room temperature. Oil-Red-O was removed and replaced with 60% isopropanol for 30 seconds.

Immunoregulation assays

To assess MSC immunomodulatory capabilities, PBMCs were stained with 5 µM 5,6-carboxyfluorescein diacetate succinimidyl ester (CFSE) (Thermo Fisher Scientific). CFSE-labeled cells were seeded on hBM-MSC monolayer stimulated (pMSCs) or not (rMSCs) with proinflammatory cytokines (IFNγ and TNFα). PBMCs were stimulated by adding phytohemagglutinin (5 µg/ml) to the culture medium. After 4 days of co-culture, cells were harvested, washed and analyzed by flow cytometry. Relative cell proliferation was expressed as percentage of median CFSE fluorescence of treated cells compared to cells treated with the specific vehicle.

Supplemental table

Table S1. List of patients and their characteristics.

Patients	*N* = 58
Gender	Females = 23
	Males = 35
Median age	57 [16–74]
FAB Subtypes (*n* = 57)	M1/M0 = 14
	M2= 13
	M3 = 3
	M4= 16

	M5=5
	M6/M7 = 1
	AML-MDS = 5
	Other = 1
Blood Parameters	Hb (g/dL) = 8.7 [3.16 – 12.9]
	PLT (10^9/L) = 67,500 [600; 319,000]
	RBC (10^{12}/L) = 20,050 [500; 21,800]
ELN/WHO stratification (n=51)	Good = 22
	Intermediate = 17
	Adverse =18
Induction therapy (58)	7+3 = 26
	MICE =26
	FLAI = 2
	Other = 4
HSCT transplantation (n=29)	Three-year remission =10
	Relapse within 3 years = 19

Patient ID	Gender	Age	FAB	Risk Stratification
Patient 1	M	48	M2	Intermediate
Patient 2	F	57	M2	Intermediate
Patient 3	F	69	M4	Intermediate
Patient 4	F	16	M5	Intermediate
Patient 5	F	70	M2	Good
Patient 6	M	64	M4	Adverse
Patient 7	M	22	M0/M1	Intermediate
Patient 8	M	35	M0/M1	Good
Patient 9	M	62	M1	Good
Patient 10	M	52	M4eo	Good
Patient 11	M	64	M3	Good
Patient 12	M	34	M2	Adverse
Patient 13	F	68	M0/M1	Intermediate
Patient 14	F	27	M3	Good
Patient 15	F	72	AML-MDS	Intermediate
Patient 16	F	53	M2	Adverse
Patient 17	M	61	M1	Adverse
Patient 18	F	51	AML-MDS	Good
Patient 19	M	70	M4	Good
Patient 20	F	58	M4	Good
Patient 21	M	27	M2	Good
Patient 22	M	63	M2	Adverse
Patient 23	M	34	M4	Adverse
Patient 24	F	48	AML-MDS	Adverse
Patient 25	M	67	nas	Adverse
Patient 26	F	62	M4	Good
Patient 27	M	42	M4	Good
Patient 28	M	60	M2/M4	Intermediate
Patient 29	M	72	M4	Adverse
Patient 30	M	35	M2	Good
Patient 31	M	54	M5	Adverse
Patient 32	F	68	M4	Good

Patient 33	F	45	M5	Intermediate
Patient 34	M	56	M5	Intermediate
Patient 35	F	51	M5b	Intermediate
Patient 36	M	54	M0/M1	Adverse
Patient 37	F	61	M0/M1	Intermediate
Patient 38	M	19	M4 eo	Good
Patient 39	M	19	M1	Adverse
Patient 40	M	66	M0	Adverse
Patient 41	M	60	M4/M5	Intermediate
Patient 42	F	67	M4	Adverse
Patient 43	M	68	M0	Intermediate
Patient 44	F	44	M4	Good
Patient 45	M	65	M1	Good
Patient 46	M	67	M4	Good
Patient 47	F	69	M2	Intermediate
Patient 48	M	59	AML-MDS	Adverse
Patient 49	F	60	M6/M7	Adverse
Patient 50	M	27	M0	Adverse
Patient 51	M	40	M2	Good
Patient 52	F	65	M1	Good
Patient 53	F	40	M2	Good
Patient 54	M	74	AML-MDS	N.V.
Patient 55	M	38	M2	Intermediate
Patient 56	M	57	M4	Adverse
Patient 57	F	32	M3	Good
Patient 58	M	44	M0	Intermediate

Legend: ELN: European Leukemia Network ; WHO : World Health Organization; 7+3 protocol: 3 days of anthracycline + 7 days of Ara-C; MICE protocol: 3 days of Mitoxantrone and Etoposide + 7 days of Ara-C; FLAI protocol: 5 days of Fludarabin and Ara-C + 2 days of Idarubicin; HSCT: allogeneic hematopoietic stem cell transplantation; AML-MDS: AML secondary to myelodysplasia.

Supplemental figures

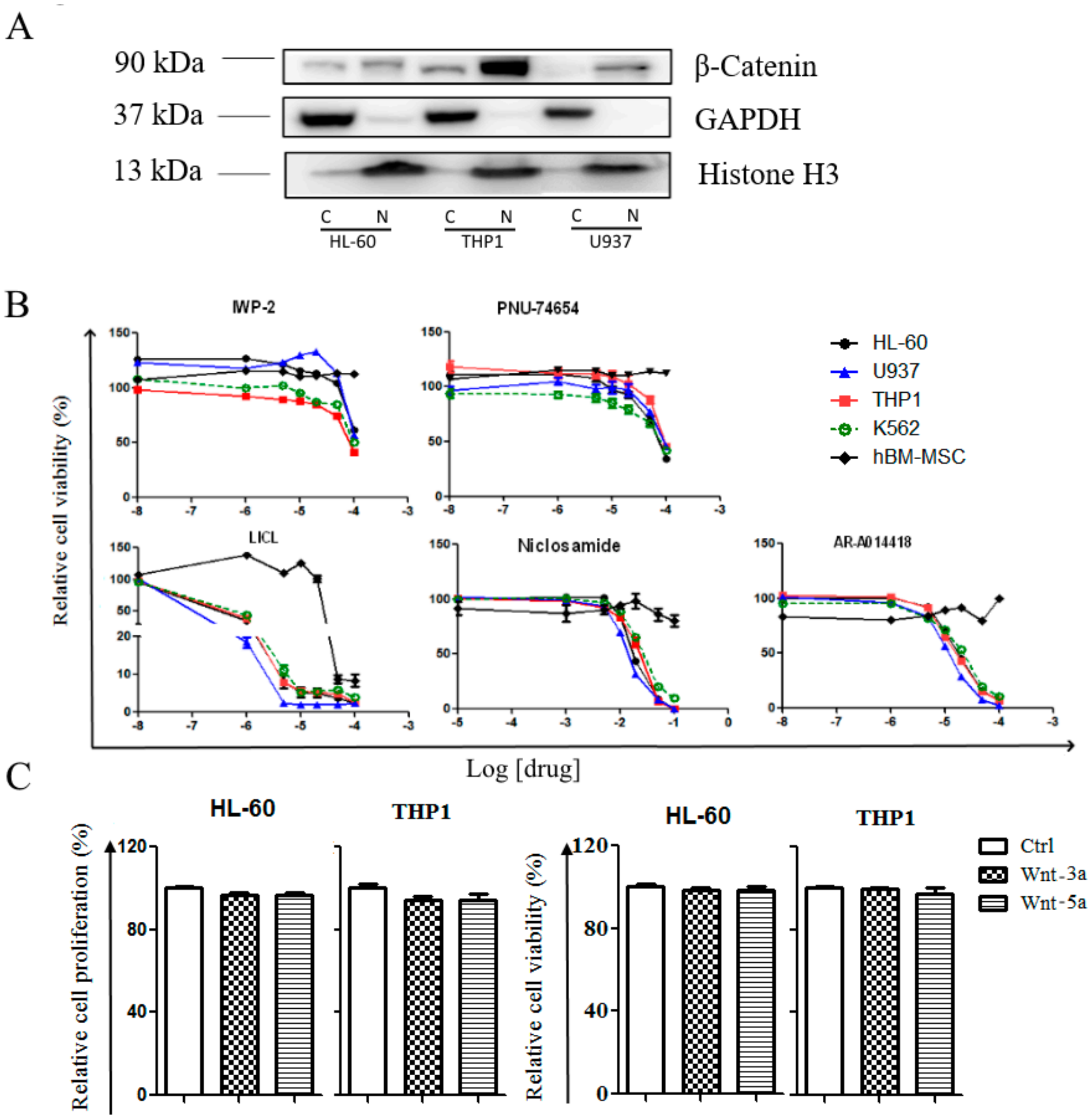

Figure S1

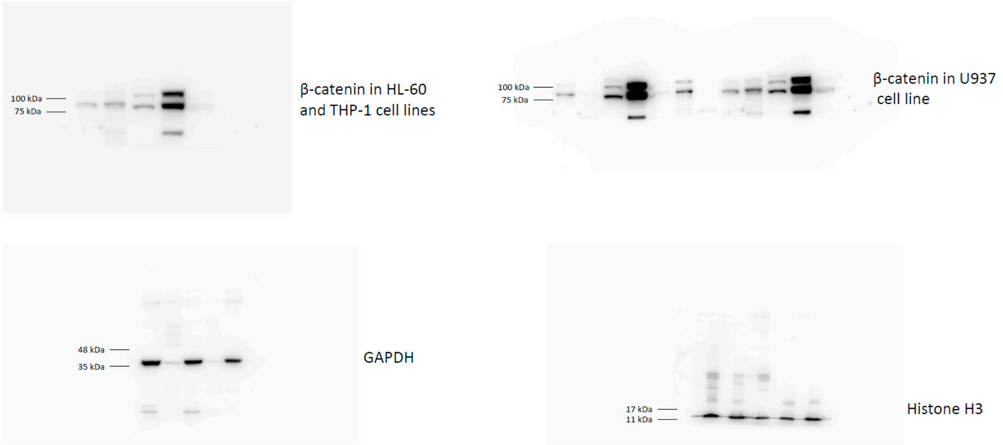

Figure S1. Wnt expression and activity in AML cell lines. (**A**) Western blot analysis of β-catenin in cytoplasmic and nuclear fraction of AML cell lines HL-60, THP1 and U937. (**B**) MTS analysis of AML cell lines treated with increasing concentrations of Wnt and GSK-3 inhibitors. Data are

representative of 4 independent experiments. (**C**) Cell proliferation and viability of AML cell lines cultured in presence of Wnt ligands Wnt-3a (25 ng/mL) and Wnt-5a (25 ng/mL).

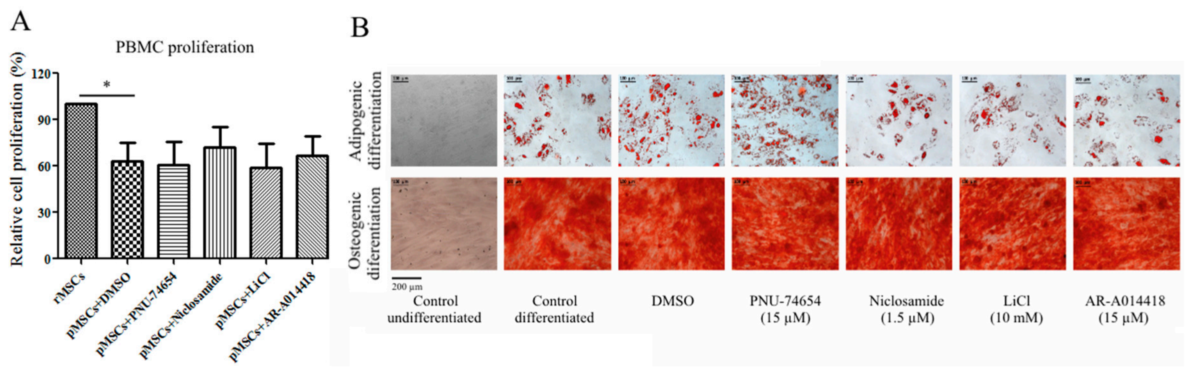

Figure S2. Differentiation and immunomodulatory capabilities of hBM-MSCs in presence of Wnt and GSK-3 inhibitors. (**A**) Oil-red-Oil (upper panel) and Alizarin red stainings of hBM-MSCs incubated for 15 days with adipogenic or osteogenic differention media, respectively. (**B**) Proliferation of activated PBMCs grown on hBM-MSCs primed with IFN-γ and TNFα (pMSCs) or not (rMSCs). Data are representative of at least 3 experiments performed in duplicate. *p<0.05.

Figure 5 (Part 1/2)

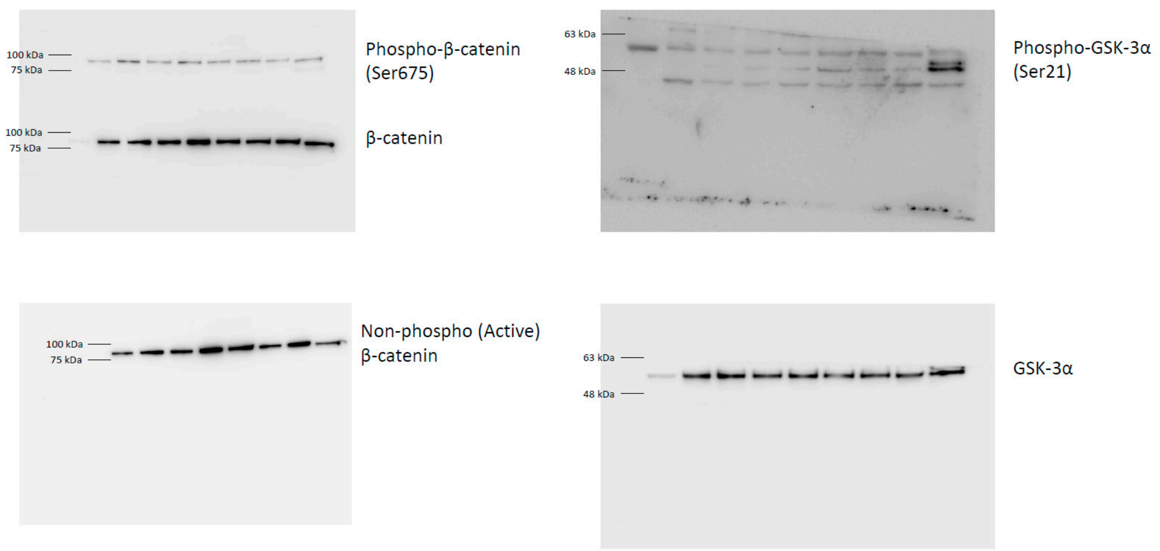

Figure 5 (Part 2/2)

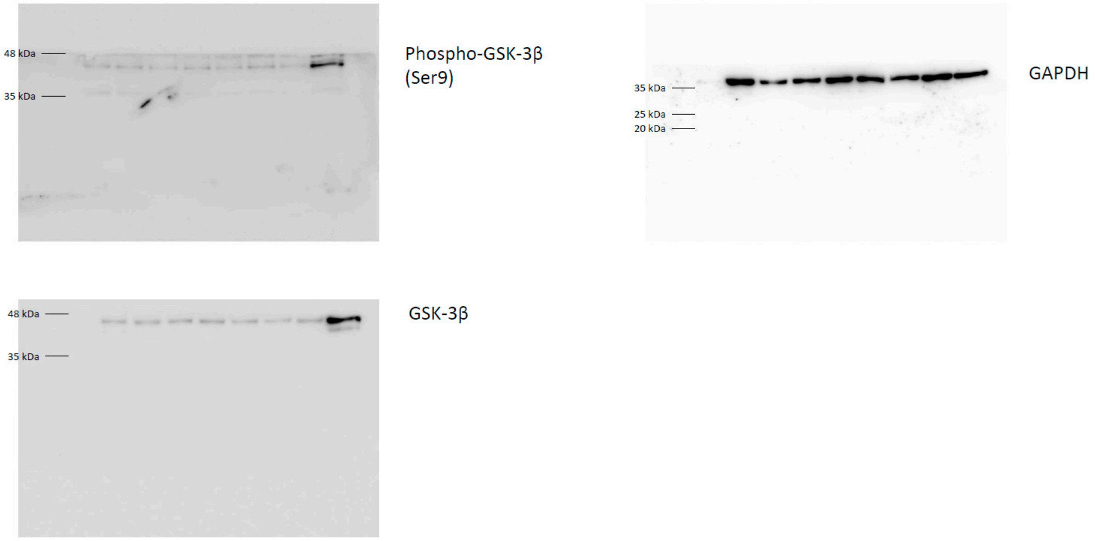

Figure S3. Original Western blot figures (Figure 5).

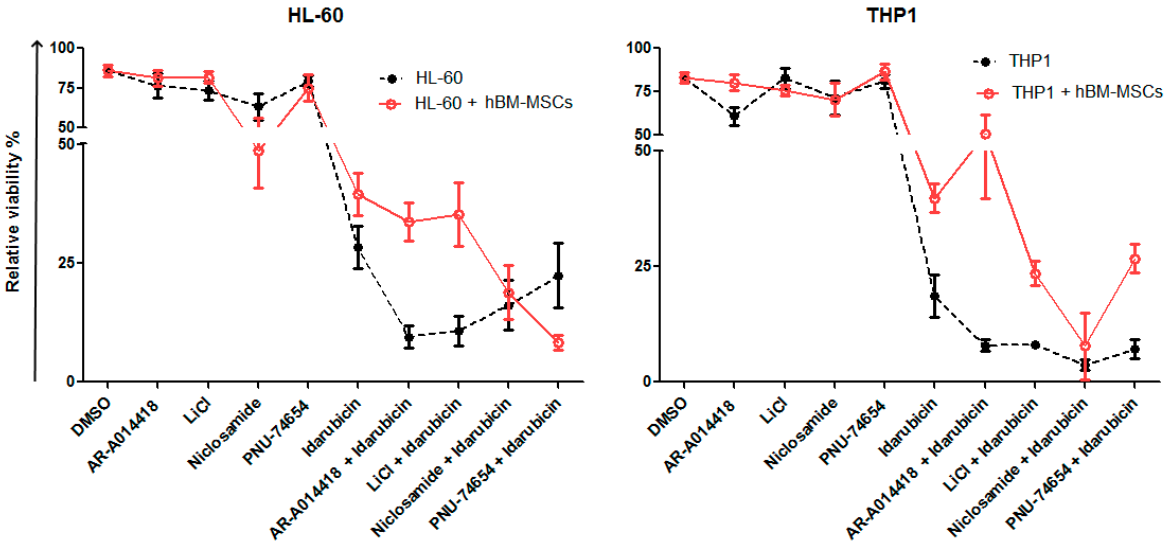

Figure S4. Contribution of Wnt modulators to AML cell lines chemosensitivity**.** AML cell lines (HL-60, THP1) were treated with Ara-C or Idarubicin in the presence or absence of hBM-MSCs and Wnt or GSK-3 inhibitors. After 4 days, cells were stained with TOPRO-3 to exclude dead cells. Viable cells (TOPRO-3 negative cells) were quantified by FACS analysis. Data are reported as mean ± SEM of at least 4 independent experiments.

Figure 6 (Part 1/4)

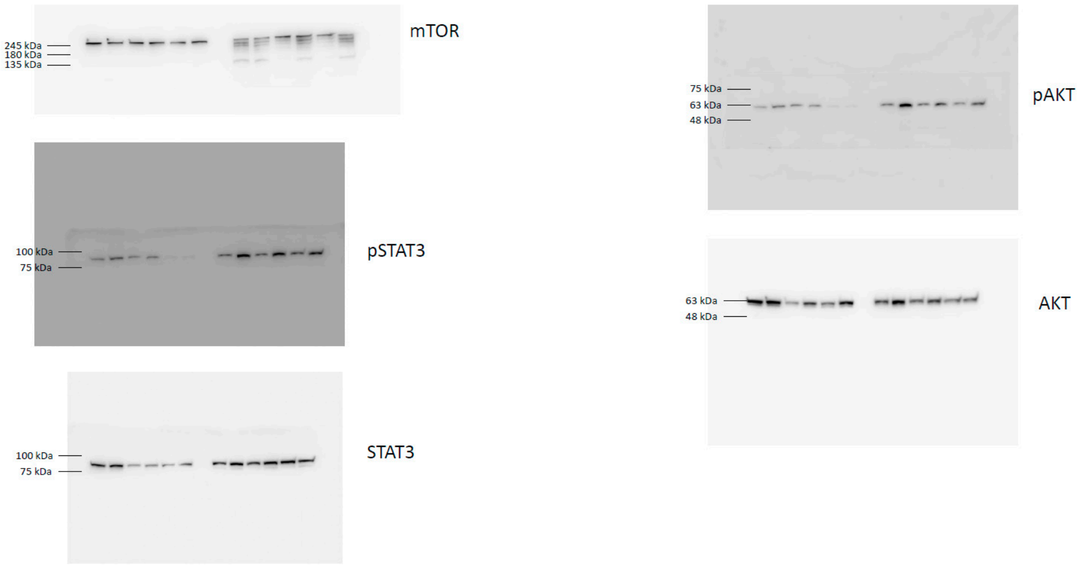

Figure 6 (Part 2/4)

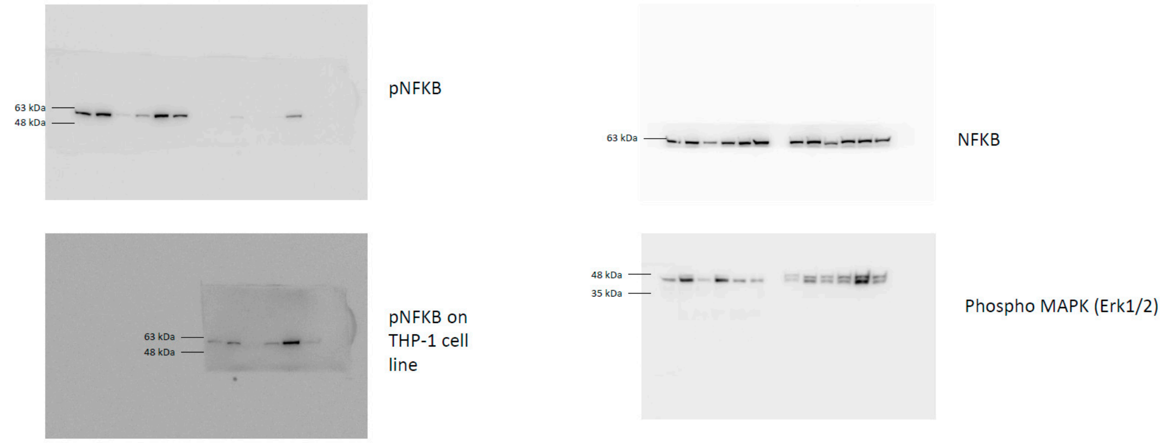

Figure 6 (Part 3/4)

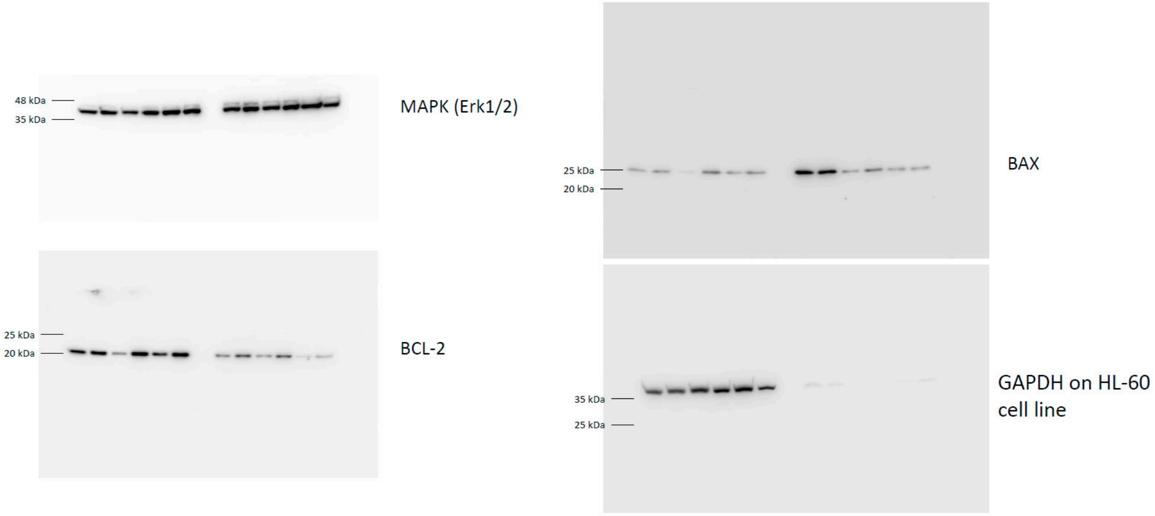

Figure 6 (Part 4/4)

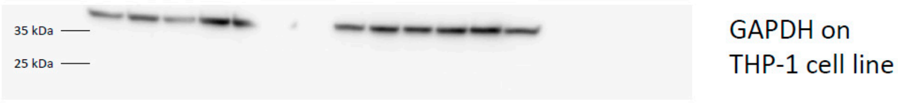

Figure S5. Original Western blot figures (Figure 6).

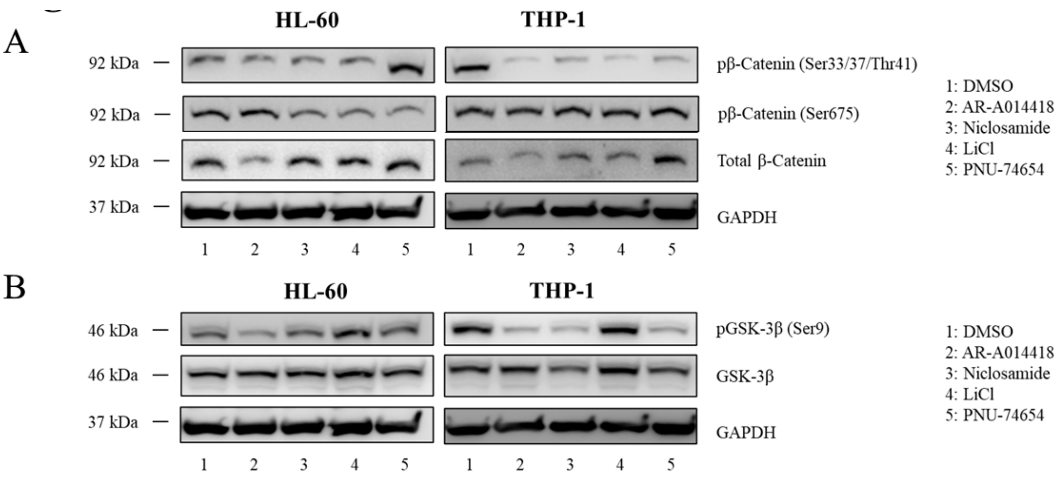

Figure S6. Representative Western blot analysis of HL-60 and THP1 cell lines treated for 48hours with Wnt or GSK-3 inhibitors, including PNU 74654 (15 µuM), IWP-2 (15 µM), Niclosamide (1 µuM), LiCl (15 mM) and AR-A014418 (15 µM). Data are representative of 2 independent experiments.
